# Design and Evaluation of a Pervasive Coaching and Gamification Platform for Young Diabetes Patients †

**DOI:** 10.3390/s18020402

**Published:** 2018-01-30

**Authors:** Randy Klaassen, Kim C. M. Bul, Rieks op den Akker, Gert Jan van der Burg, Pamela M. Kato, Pierpaolo Di Bitonto

**Affiliations:** 1 Human Media Interaction, Faculty of Electrical Engineering, Mathematics and Computer Science, University of Twente, P.O. Box 217, 7500 AE Enschede, The Netherlands; h.j.a.opdenakker@utwente.nl; 2 Centre for Innovative Research across the Life Course, Faculty of Health and Life Sciences, Coventry University, CV1 5FB Coventry, UK; ac2658@coventry.ac.uk; 3 Gelderse Vallei Hospital, P.O. Box 9025, 6710 HN Ede, The Netherlands; burgg@zgv.nl; 4 School of Computing, Electronics and Mathematics, Faculty of Engineering, Environment and Computing, Coventry University, CV1 5FB Coventry, UK; pam@pamkato.com; 5 Grifo multimedia Srl, Via Bruno Zaccaro, 19-70126 Bari, Italy; p.dibitonto@grifomultimedia.it

**Keywords:** digital coaching, diabetes education, serious gaming, self-management, user evaluations

## Abstract

Self monitoring, personal goal-setting and coaching, education and social support are strategies to help patients with chronic conditions in their daily care. Various tools have been developed, e.g., mobile digital coaching systems connected with wearable sensors, serious games and patient web portals to personal health records, that aim to support patients with chronic conditions and their caregivers in realizing the ideal of self-management. We describe a platform that integrates these tools to support young patients in diabetes self-management through educational game playing, monitoring and motivational feedback. We describe the design of the platform referring to principles from healthcare, persuasive system design and serious game design. The virtual coach is a game guide that can also provide personalized feedback about the user’s daily care related activities which have value for making progress in the game world. User evaluations with patients under pediatric supervision revealed that the use of mobile technology in combination with web-based elements is feasible but some assumptions made about how users would connect to the platform were not satisfied in reality, resulting in less than optimal user experiences. We discuss challenges with suggestions for further development of integrated pervasive coaching and gamification platforms in medical practice.

## 1. Introduction

Self-management [1] is of key importance in the successful treatment of patients with chronic conditions, such as Type 1 diabetes (T1D), a condition in which the patient needs daily insulin treatment because their body fails to produce this hormone. In contrast to Type 2 diabetes, T1D is typically diagnosed at a young age when patients are still dependent on their parents who are responsible for managing their child’s health condition [2]. Children are supposed to gain more responsibility for their T1D care when they become adolescents. Goals for the management of T1D include achieving optimal glycemic control, avoiding acute complications, and minimizing the risk of long-term microvascular and macrovascular complications. Self-monitoring of blood glucose (BGM), either by using a blood glucose meter or using a system for continuous blood glucose monitoring [3] is one of the most important activities in managing diabetes. Research repeatedly shows that adherence to BGM is linked to glycemic control in pediatric T1D [4,5,6,7]. That is, the more frequently the patient measures their blood glucose (BG) using reliable, certified blood glucose meters [8], the more their BG will be within acceptable and appropriate levels. International studies show that metabolic control is unsatisfactory in many adolescents with T1D [7,9]. Despite advances in technologies that support BGM, frequent measuring is still a burden for many children and adolescents [10,11,12]. Adherence, i.e., the degree to which the person’s behavior corresponds with the agreed recommendations from a health-care provider, is a key factor in the successful treatment of chronic conditions. Even when patients have good knowledge about treatment adherence, their actual practice of adherence is often less than ideal [13]. One of the factors that is often underestimated in T1D management is the burden of the disease in everyday life. The patient is constantly required to perform actions and make decisions in their daily diabetes routine. Examples are: finger pricks for glucose measurements, insulin injections, replacing needles for insulin pumps or subcutaneous glucose sensors, carbohydrate counting, and regime adjustments for physical activities or sports, for sick days, for parties, etc. These requirements interfere with normal life, especially for adolescents who want to be perceived as “normal”. Aversion to these compelling actions often means that children and adolescents do not achieve a good metabolic control. Research on the motivations for and causes of non-adherence to diabetes care regimens among adolescent diabetes patients points to the contrasting views on adherence between patients and providers. Where providers typically view adherence in pursuing optimal glycemic control and health outcomes, patients have other perspectives [11]. Hence, one of the adherence challenges with T1D is motivating and helping these patients measure their BG at regular times every day in such a way that it is least invasive using methods and tools popular among this target group. Digital coaching systems that monitor the users’ BGM send reminders and motivating messages to support the patient adhere to a personalized and clinically appropriate medical regimen. However, to function properly these systems need regular data from the user, which often requires additional actions on the part of the user; e.g., upload glucose data. The challenge is then to motivate the patient to keep using these tools on an ongoing regular basis. How can technology help these patients to make it a bit easier to learn to live with their condition and to adhere to a medical regimen as long as there is no cure?

The pervasive gamification and coaching platform presented here is the product of the EU Horizon 2020 PERGAMON project (No. 644385) in which a number of developments came together that exploit opportunities offered by the availability and acceptance of new technologies in e/m-health: reliable wearable sensors, mobile apps that support self-management and lifestyle behaviour change, digital coaching, serious games and gamification to enhance healthcare education, and Patient Web Portals connected to Personal Health Records, that allow patients to upload personal data and receive personalised guidance from caregivers. The platform integrates the complementary potential offered by the widespread use of wearable sensors and mobile devices and the popularity of games and social networks delivered on a secure and authorized portal that supports patients and their caregivers in self-management of their chronic condition. Reviews of studies in the development and evaluation of the use of each of the above mentioned component technologies show besides (potential) positive impact on diabetes care and opportunities, that there are also challenges that demand a more integrated solution [14].

The management of diabetes is complex and patients need personalized advice and medical control by a pediatrician or diabetes nurse. The growing number of diabetes patients worldwide place a heavy burden on medical budgets. Patient web portals are increasingly set up and maintained by hospitals to support self-management and improve communication between patients and their medical caregivers, as one of the most important predictors of adherence [13]. A review by Osborne et al. showed that they have a positive impact on management for diabetes care [15]. The communication between health provider and patient could benefit from integration of these web portals with the mobile technology and “real-time” sensoring available on smartphones.

What can we say about quality, engagement and effect in terms of adherence when treatment is supported by mobile diabetes apps? While a wide selection of mobile apps is available for self-management of diabetes, some specifically designed for patients with T1D [12], some with a virtual coach [16,17], current research suggests that most do not meet basic requirements for medical applications [18]. From a medical perspective a main finding from a review by Chomutare et al. [19] is that a critical feature strongly recommended by clinical guidelines, namely, personalized education, is not present in current applications. Although mobile health apps have great potential for improving chronic disease care, they face a number of challenges including lack of evidence of clinical effectiveness and lack of integration with the healthcare delivery system. There is a clear need for formal evaluation and review of potential threats to safety and privacy [20]. Brzan et al. [18] reviewed 65 apps for diabetes and concluded that 56 of these apps “did not meet even minimal requirements or did not work properly”. They report on a qualitative study in young adults with the objective to explore their experiences with apps that aim at health behavior change and their willingness to use these apps. Many participants in their study “were not motivated enough to regularly and precisely use the apps in making healthy lifestyle changes”, a recurring issue in many studies. Boyle et al. [21], reporting on a survey of patients seen at a hospital diabetes clinic in New Zealand found so many concerning issues that they concluded that there is a need for an app assessment process to raise confidence in the quality and safety of diabetes management apps in diabetes patients as well as in healthcare providers.

Although short term user studies often show promising results in terms of adherence to medical treatment there is no evidence of maintenance of high adherence in the long run. According to self-determination theory, it may take many months before external motivation becomes internalized into daily routine behaviours [22]. Hence there is a need for ways to motivate patients with T1D to manage and adhere to their treatments on an ongoing basis.

Gamification techniques have been introduced as possible means to motivate patients to sustain adherence to medical treatment [23]. Gamification is using elements of game design [24], i.e., points, leader boards, levels, competitions, rewards, achievements, mini games, goals, experience points, rules, narrative, graphics, imagination, role identification, or setting step-wise challenges in pursuit of a goal. Use of games or gamification in health behaviour change programs might thus be a way to intrinsically motivate users to expose themselves to and continually engage with these programs (Baranowski et al. [25], Thompson et al. [26], Cugelman [27]). In serious games, elements of game design [28,29] are used to help the user learn to reach non-game goals. The ultimate goal is to foster intrinsic motivation for learning and maintaining desired behaviours [30].

Educational games in diabetes care dates back to the 90s. Lieberman [31] describes 14 diabetes self-management video games. The games typically involve players in problem-solving and decision-making in simulations of diabetes self-management, usually by asking players to balance food intake and insulin injections to keep a game character’s blood glucose within a normal range. An important feature of these games is that they provide practice through rehearsal and show cause and effect, while also providing basic information about diabetes self-management. There are many games that teach the relationship between food (carbohydrates), plasma glucose level, exercise, and insulin dose [32,33], or that focus on learning the number of carbohydrates in drinks, snacks and meals [34]. Several studies using randomized controlled trial designs, e.g., Brown et al. [35] and Fuchslocher et al. [36], showed that diabetes related content explicitly presented in games improves diabetes self-management in T1D. In a study by Lieberman [37] a diabetes game reduced diabetes-related urgent and emergency visits by 77% after young patients had the game at home for six months, compared to no reduction in clinical utilization in a control group of young patients who took home an entertainment video game that had no health content.

In a special issue on Games for Diabetes, Theng et al. [38] provide a review of evidence on the efficacy of video games and gamification in diabetes self-management (not specifically targeting, but including T1D). The duration of most of the ten studies was short with small sample sizes of those that studied patients aged between 8 and 16 years old. All the interventions targeted behavioural changes to promote healthy behaviours among the study participants. Video games were found to be effective tools for education while gamification and virtual environments increased intrinsic motivation and positive reinforcement. Remarkably, their study did not find any research specifically targeting medication adherence as part of the behavioral change process.

Charlier et al. [39] reports a review of randomized controlled trials (RCTs) assessing efficacy of serious games in improving knowledge and self-management in young people with chronic conditions. From 9 studies the general conclusion is that educational video games improve knowledge and self-management. Johnson et al. [40] identify potential advantages of gamification from existing research and conducted a systematic literature review of empirical studies on gamification for health and well-being, assessing quality of evidence, effect type, and application domain. They conclude that due to the relatively small number of studies and a lack of studies that compare gamified interventions to non-gamified versions of the intervention, it is hard to draw general conclusions about the efficacy of gamification in digital health interventions. Deacon and O’Farrell [41] and Sardi et al. [42] come to a similar conclusion: there is still a lack of valid empirical evidence that support the use of gamification strategies employed in e-Health.

Some studies indicate that gamification of BGM has positive effects on BGM in adolescents with T1D [43,44]. In the mobile game, DIAL, a group of children (8–18) were given a mobile device with an integrated motivational game in which the participants could guess a BG level following collection of three earlier readings. In a 4 week experiment, the game group sent significantly more glucose values to the platform than the control group that did not have the game. Use of a motivational game appears to increase the frequency of monitoring, reduce the frequency of hyperglycemia, improve diabetes knowledge and may help to optimize glycemic control. Follow-up research in a RCT among adolescent T1D patients over 12 months showed that app usage diminished over the trial. On average, 35% (16/46) of the participants were classified as moderately or highly engaged (uploaded glucose data 3 or more days a week) over the 12 months. S. et al. [14] suggest exploring the utility of integrating mobile applications for T1D support into routine clinical care to facilitate more frequent feedback.

Does access to video games or the possibility of making progress in a video game work as a reward for regular glucose measuring and control? In a study by Klingensmith et al. [45] acceptance of a system that connects a blood glucose meter with a Nintendo game was assessed in a sample of children, adolescents, and young adults with T1D. Users receive reward points that can be transferred from the meter to the video game, allowing access to new levels of play and mini games. Rewards are based on frequency, timing and results of blood glucose testing. Healthcare providers can also set personalized target ranges in the meter to help patients reach glucose goals. The majority of healthcare providers agreed that the coupled system would solve a problem in diabetes management, and that it would motivate patients to test their blood sugar. They observed an increase of use in the home situation compared to the lab situation because users wanted to have more advanced games [45].

Besides monitoring and feedback, personal coaching and goal-setting and learning how to cope with situations that impact blood glucose level, social support is an important strategy to help young diabetes patients to adopt healthier habits. A rationale for using games for serious purposes like health is their ability to motivate and facilitate social encounters [28]. Children like to play together even when playing single player games. The effect of social support on users’ motivation to use mobile apps appears to be mixed. Some are motivated by competition among peers, where others feel that sharing data and results introduces too much competition [46]. The gaming framework of Chomutare et al. [30] focuses on completing self-management tasks, rather than rewards or penalties. They emphasize the value of cooperation, social comparison and a focus on positive achievements as core game elements, limiting the extent of competition. Social media in combination with serious games are suggested as a beneficial platform for self-management of T1D by the younger age groups [47]. Social media are popular among adolescents. They can interact and build communities dedicated to specific games. Social media allow T1D patients to share their game experience with everyone; not only with other patients with diabetes. A disadvantage of such an open community is the lack of a quality control of the content which is mostly provided by the community itself [47]. This is one of the reasons we chose an environment with authorized users where content is moderated by the care institute.

The above review presents a state of the art of existing applications. The PERGAMON platform (introduced in Section 1.1) integrates techniques and design decisions that we find in the applications discussed above. A system that integrates all the diabetes educators by integrating gaming and coaching in a clinical setting through a principled design would overcome many of the shortcomings of the partial solutions in reviewed systems (e.g., no personalization, not specifically focused on medical adherence).

### 1.1. The PERGAMON Platform

The PERGAMON platform that we present here is a gamification platform that integrates educational gaming and coaching. It gamifies self-care-related activities so users are rewarded for making progress in the game world. The virtual coach can keep track of users’ daily BGM, physical activity, food intake and send reminders and motivational messages when the user forgets to follow recommendations agreed upon with their healthcare professional. The coaching system can reward behaviours that correspond to personalized goals. The platform provides a secure way for message exchange and data sharing between patients, their peers and their caregivers.

The platform integrates game elements and virtual coaching with a well designed system of main game and mini games based on an ontology of tasks, sub-goals and goals. Moreover, contrary to many systems medical content and coaching messages are tailored and personalized.

The aim is to see how gamification and digital coaching can be integrated into the continuous care of patients with chronic conditions, with the aim to integrate the findings in existing Patient Web Portals that support care of patients with T1D. Involving young patients with T1D and their caregivers in the development process was part of the development and research process. Young people and adolescents in particular have different requirements for medical devices than adults [48]. They also have different preferences for types of games and gamification methods [49].

In this research we investigate if the PERGAMON framework does support the specification and implementation of a pervasive serious gaming and coaching system for specific target groups. We demonstrated the answer by developing the system for young children with diabetes in a clinical setting. Next to this question, this research tries to investigate if patients do like the PERGAMON platform, how they use the different functions and the games of the platform, and if the integration of the components motivate educative game play as well as self-management of diabetes (in particular adherence to blood glucose monitor use).

After we discuss design principles and the design of the PERGAMON gamification platform, we present preliminary pilot study results obtained with experiences with the platform “in the wild”, that is, with young patients with T1D under supervision of paediatricians and diabetes nurses in a Dutch hospital. The aim of the evaluation was to see whether patients had problems using the system and how they assessed the games and the coaching. Such usability and experience evaluations are a prerequisite for future evaluations that measure the impact of the platform in terms of medical outcomes.

## 2. Design Principles for the Gamification Platform

The design of the gamification and coaching platform adheres to basic principles of healthcare, design principles for serious gaming as well as design principles for behaviour change support systems. Care values were considered in determining design, especially regarding what techniques and content the system implements, how the system communicates with the user, and how the system treats personal information, regarding security and privacy issues. The gamified platform involved introducing intelligent systems to users that could to some extent take over the role of human caregivers. This raised issues of responsibility for protecting care values [50]. In our view, a system can never take away the responsibility from the human user (e.g., the care provider, or the patient). On the contrary, the design should aim at supporting the patient in becoming a responsible person in their self-care by providing the patient the means, along with supportive motivation, to make a well-informed decision. Tailoring to the individual user and his or her social and intellectual abilities was therefore an important requirement for the design. The aim of technology for self-management in healthcare is not to completely replace the human care system but to support and enhance it. The PERGAMON system was designed to be integrated in the care and treatment of young patients by the medical caregivers, e.g., pediatricians, diabetes nurses, and informal carers. Patient’s credibility rating of the system depends on how trustworthy the medical experts are that provide input into the design of the system. Educational goals and targets of diabetes self-management formulated by diabetes care organizations were used to drive the content and design of the system as a whole as well as for the specific themes of the games in the system. Specifically, these goals (diabetes educators) were balancing energy through medication intake, healthy eating, being physically active, learning how to monitor blood sugar levels, coping with high and lows of BG and how to reduce risks associated with BGM. The system guides the individual and establishes together with the user specific, measurable, attainable, realistic, and timely (SMART) goals [51].

The design of a behaviour change support system for health interventions [52] involves the implementation of several behaviour change techniques, such as feedback and monitoring, reward and threat, and social support. A behaviour change technique is a component of an intervention designed to alter or redirect causal processes that regulate behaviour [53]. They are based on socio–psychological behaviour theories, e.g., Goal-Setting [54], Self-Determination Theory [55]. Behavior change techniques (BCTs) were integrated into the Persuasive System Design Model (PSD) for behaviour change support systems [52] that we use to describe the system’s design. The PSD model has four categories: primary task support, dialogue support, system credibility support and social support, each with a number of design principles (to be distinguished from the moral principles of health care). Key aspects of BCTs were integrated, e.g., self-monitoring, rewards or provide information about others approval. Although Abraham et al. [53] presents 26 BCTs, the principles in the category of System Credibility Support in the PSD model do not occur in the BCTs listed. This is understandable because the PSD is intended for the design and evaluation of a technical system, not human beings per se. These systems function as more or less autonomous entities in situations where the medical care giver is not immediately present for the user. Also, by their very nature, technical systems have —seen from a design perspective— abstract users so that for the system designer tailoring and personalisation become a concern (partly solved by explicit user models and use of persona in the design process) where treatments by human caregivers and coaches (different from rule following machines) are tacitly assumed to tailor their treatment to the individual patient.

Some of the design principles of the PSD model are implemented in our system by means of specific game elements (which encompass game mechanics, themes, game characters, challenges, rewards) from game design [28]. For example, the principles of reduction and tunneling in the PSD model are materialized by dedicated learning tasks in different mini games and by distinguishing different reachable goal levels. Other principles are implemented in actions of the virtual coach: praise, rewards, reminders, suggestions as well as by various game elements. Similarity and social role are materialized by game elements that aim at identification with the main character in the game as well as by the use of a similar character for the virtual coach.

The educational goals for diabetes care (e.g., the BCT provide information on consequences [53]) are implemented in the adventure game, called the Tako Game, in particular in a number of integrated educational mini games and by educational elements such as videos. The Tako Game is a “pervasive” game in the sense that it expands the “magic circle” of play [24,28]: the border between game world and reality is blurred by making daily self-management activities relevant in the game.

## 3. PERGAMON: A Framework for Gamification, Real-Time Sensoring and Virtual Coaching

The TIKI TAKO system for young T1D patients is an instantiation of the general PERGAMON framework. The PERGAMON framework allows the creation of behaviour change support systems [56] that combine serious gaming (a main game and mini games), virtual coaching and real-time monitoring of activities in daily life via sensors.

The architecture of the PERGAMON framework is a Service Architecture. The system is not a set of isolated applications communicating based on the integration of the different parts of the application. The framework is organized in a collection of services that can published on a communication infrastructure. These services can be used by multiple applications. The main services in the PERGAMON framework are; (1) Saving and sharing data from the sensor network (Glucometer data; Pedometer data; Insulin Pen data), (2) Saving and sharing user state (e.g., achieved goals, badges), (3) Analysis of the data coming from sensors (i.e., Pedometer, Glucometer, Insulin Pen) and (4) Virtual Coach services. The infrastructure enables the various “consumers” of data services to query and access the available information on heterogeneous information systems through the exchange of messages. In particular, for the sensor network a form of hardware abstraction is needed, which involves having a custom plugin for each device that will be supported. The architecture of the PERGAMON framework supports the implementation of real-time behavioral change techniques (see Section 2) such as real-time feedback and monitoring of behavior of users.

**Figure 1:**
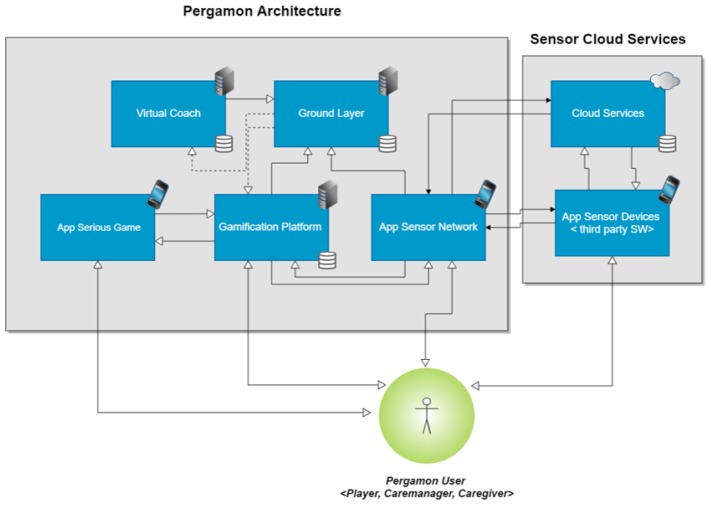
The PERGAMON architecture.

A typical system created with the PERGAMON framework consists of a web application (website), an Android application for gathering data from sensors, an Android webapp to view the PERGAMON website via an Android device and an Android Unity application for the games. The framework consists of five key components (Figure 1):

- Ground Layer (the central data hub)
- Sensor Network
- Gamification Platform
- Serious Game (the main game and the mini games)
- Virtual Coach

In the following subsections, we will discuss the different components in detail.

### 3.1. Architecture of the PERGAMON System

Figure 1 presents the architecture of the PERGAMON framework. The Ground Layer has a central place: it stores data from the other components and makes the data available to all connected components via Application programming interfaces (APIs). The connected components are the (i) virtual coach; (ii) serious game(s); (iii) gamification platform; and (iv) the sensor network.

Communication between the different components and the Ground Layer are over secure HTTPS connections and basic authentication is needed to access the RESTful APIs (Representational state transfer (REST) or RESTful web services is a way of providing interoperability between computer systems on the Internet.). The other components in the PERGAMON framework can read, write, update and delete data in the Ground Layer.

### 3.2. Sensor Network

The Sensor Network component is responsible for the connections between the different sensors used to monitor the users of the system and the PERGAMON framework. The Sensor Network is running on an Android device (smartphone or tablet) and turns it into a sensor hub to which different devices can be connected.

**Figure 2:**
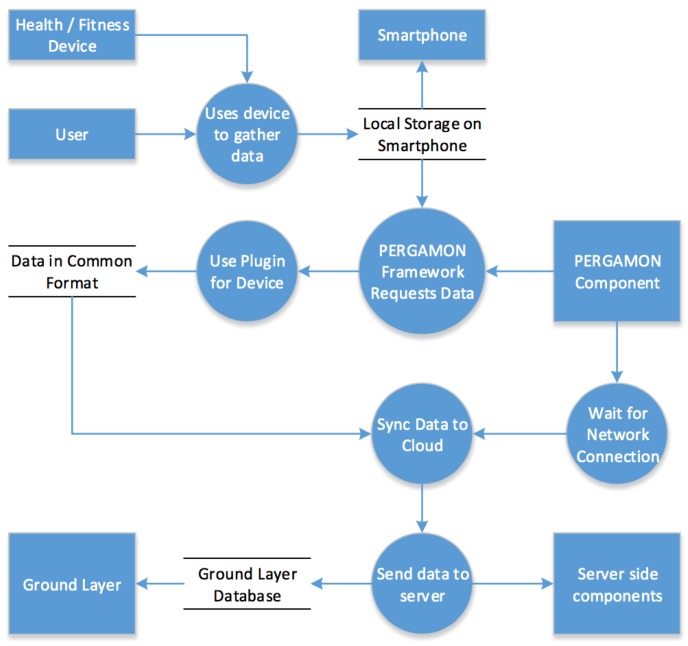
The data flow of the Sensor Network.

Devices are made compatible with the PERGAMON Sensor Network via a plugin system. Each device must be categorized into a device type, such as “Pedometer”, and all devices of that type must use a plugin to provide data to the server to store in the Ground Layer database in a common format. Each plugin written for the Sensor Network can have its own behaviour for obtaining the data from a device. Plugins simply operate via a common request and reply system where the custom behaviour required to interface with each device is contained purely within the plugin and hidden from other PERGAMON components. It is through this plugin architecture that the Sensor Network allows the PERGAMON framework to work with a large range of devices, as well as allowing devices to be easily supported in future. Once data is gathered from the devices connected to the Sensor Network, the data must be pushed up to the cloud once a network connection is available. Data will be held on the device and synchronised with the Ground Layer via a connection to the servers when possible, in order to allow the PERGAMON app to gather data while offline. Once the data is confirmed to be stored in the Ground Layer, the Sensor Network’s role in the PERGAMON framework is complete until more data is gathered. Figure 2 presents a schematic overview of the data flow of the Sensor Network.

**Figure 3:**
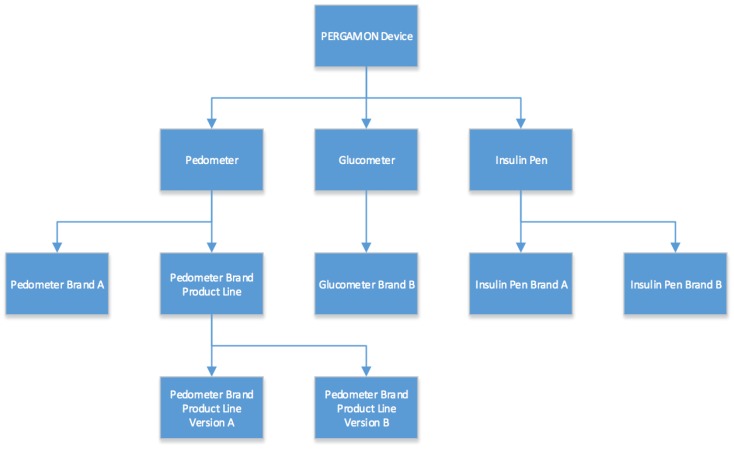
The architecture of the Sensor Network.

The architecture of the Sensor Network will follow a classic object oriented approach (see Figure 3). Every device will be a “PERGAMON Device” in the system, and as such all devices will have the common functionality associated with it. PERGAMON Devices will implement the very basic behaviour of request and reply handling, as well as containing the identifier for which device this class represents. On the next level in the architecture, different types of devices can be found. The three initial types are “Pedometer”, “Glucometer” and “Insulin Pen”. These classes define in which data format the devices will be expected to return data. Every device will fit one of these categories and more categories can be added if needed.

The lowest levels in the class hierarchy will be the individual device plugins. They represent actual physical devices instead of categories into which they should fit. They will define the actual behaviour that is executed when the functions defined by parent classes are called.

The architecture of the Sensor Network makes it possible to add new devices and services in an easy and modular way. Compatible sensors and services (currently the Google Fit service, Mi Fit band and Mi Fit band 2 and Menarini blood glucose sensors are supported) connected to the Sensor Network will automatically record data. The Sensor Network will gather the recorded data, process the data and push it to the Ground Layer component. The Sensor Network also provides the possibility for users to enter data manually, such as food intake, mood or sleep.

### 3.3. Gamification Platform

The third component of the PERGAMON framework is the Gamification Platform. Goals in the platform can be defined and created by a game designer using a platform designer’s interface. The main gamification elements in the PERGAMON platform are Tasks and Goals. A task is the basic unit, and corresponds to a single action that can be performed by the user. When a task is complete, the user will receive a certain number of points. A goal is a set of different tasks. The completion of a goal will give the user a bonus score, translated into points. Tasks can belong to the following activities:

- System’s activity, i.e., all the activities concerning the PERGAMON framework (User Profile, Social Hub, Food Diary, and so on).
- Sensor Network, i.e., all the activities concerning the use of the glucometer and pedometer.

**Figure 4:**
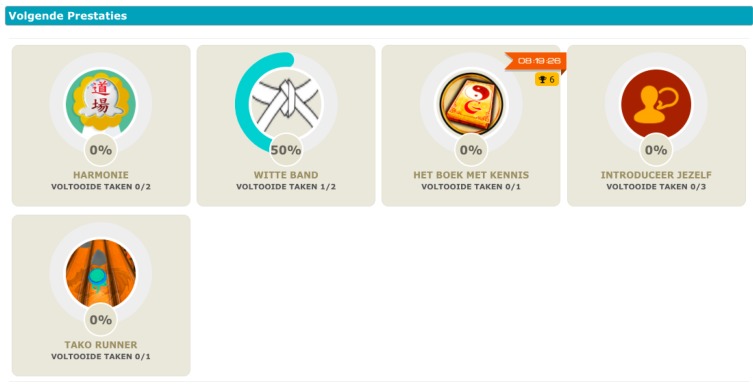
Gamification platform, Goals.

**Figure 5:**
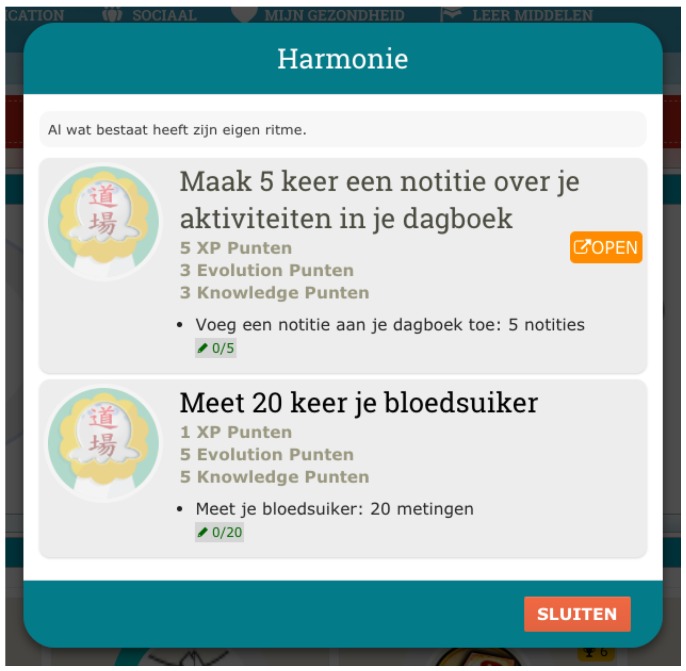
Gamification platform, Tasks.

All the activities in the PERGAMON framwork are gamified, e.g., each interaction with the system will be associated with a score. Users will receive immediate feedback by completing tasks, and gaining scores. A progress bar will indicate the goal percentage of accomplishment showing the progress the user has made (see Figure 4 and Figure 5). The difficulty of the goals will increase when the user advances in using the system. Goals can be mono-thematic (e.g., all about diet or physical activity) or multi-thematic (e.g., “A perfect healthy day”, that includes diet, physical activity, and therapy management).

Users can challenge themselves, deciding to accomplish a task within a certain period of time, receiving, as a consequence, a bonus score, or they can simply conduct their normal life, and accomplish tasks unintentionally as part of their daily routine, while their accomplishments are recorded by the sensor devices from the Sensor Network. Points are associated with both tasks and goals; each task will have its corresponding score, while a goal will give a bonus score as reward. In fact, once a goal is accomplished, users will receive a bonus percentage of points. The gamification platform manages three kind of points:

1. Experience: this type is associated with the general leader board, and allows unlocking special objects in the serious games.
2. Knowledge: this type is associated with the serious game environment and allows skipping between levels
3. Evolution: this type is associated with a specific instance in the gamification.

These three different kinds of points are needed to continue playing the game and are used to create a leader board to share this with friends.

### 3.4. Games

The PERGAMON framework supports two different kinds of games, the main game (called *Tiki Tako*) and mini games. The main game is an adventure/puzzle game that can be played over a longer period of time. Users have to solve puzzles and levels to make progress in the game. Points (experience, knowledge and evolution points) earned by completing tasks and goals, either in the real world or in the game world, are needed to continue the main game. In this way the main game is linked to the tasks and goals from the real world and to the other components of the PERGAMON framework. Mini games are played in a shorter timespan than in the main *Tiki Tako* game. They are designed to communicate certain educational objectives related to the chronic condition by means of game metaphors. Users are required to play the mini games that are played outside the *Tiki Tako* game to make progress in the main game. The games in the PERGAMON framework are developed in the Unity game engine using the PERGAMON API.

### 3.5. The Mystery of TIKI TAKO

**Figure 6:**
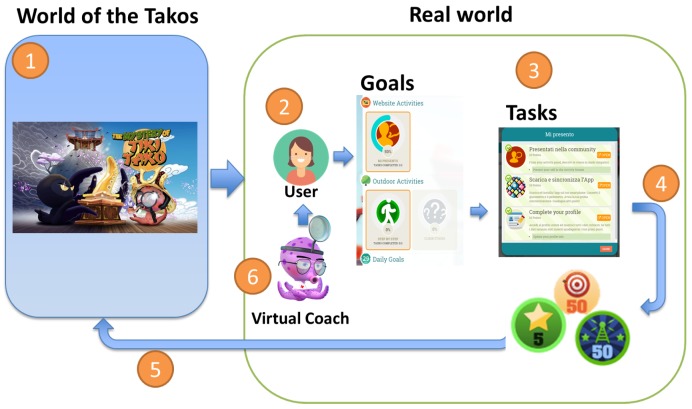
Main characteristics of the *Tako Game* (see Table 1 for explanation).

The TIKI TAKO system for young T1D patients is an instantiation of the general PERGAMON framework. The characteristics of the TIKI TAKO system are presented in Figure 6 and Table 1. The Ground Layer of the TIKI TAKO system supports the storage of data and measurements about physical activity, glucose measurements, insulin intakes, and food. The Sensor Network of the TIKI TAKO system supports different channels and devices that are able to collect information about daily life activities via sensors. It supports the Google Fit service which collects information about physical activity (number of steps and activity intensity). Other sensors compatible with the Google Fit service could be used in the TIKI TAKO system to also collect data about physical activity. We used the Google Fit ©Pedometer (as a service on the smartphone itself, or on an Android Wear smartwatch) and the Xiaomi Mi Fit Band ©(in combination with the Mi Fit app) as sensors. Also the services and devices of iHealth are supported to collect physical activity or blood glucose measurements. Menarini ©blood glucose sensors are also supported to measure blood glucose. The Sensor Network also allows for manual input of information about diet, insulin and sleep.

Within the Gamification Platform five types of goals and tasks for young T1D patients have been implemented:

- “My Sugar” tasks relate to measuring blood glucose, reinforcing the relationship between diet and glucose levels, education about hypo and hyperglycemia or taking notes regarding insulin use.
- “Physical activities” are tasks related to diabetes and physical activity and sports such as setting a number of steps as a daily goal or learning about how to prepare to engage in sports.
- “Active life” tasks support the user to self-manage their diabetes in a more introspective way rather than overtly directing their activities. Examples are writing a to-do list to plan activities in a day or reviewing one’s activity or BG data.
- “Website Activities” are tasks related to the use of the system and the website such as creating a profile or installing applications on the smartphone.
- “Mystery of TikiTako” tasks are related to the main game of the TIKI TAKO system such as how to continue in the game or how to finish a mini game.

The Serious Game component of the TIKI TAKO system consists of the main “Mystery of TikiTako” game and seven mini games. The main game is an adventure and puzzle game in which the player takes on the role of an investigator who tries to solve a mystery. The investigator, the main character in the main game, also has T1D. The player progresses in the game by successfully directing the main character to control his diabetes and solve puzzles through the mini games that have educational goals, such as how to do insulin injections or how to balance blood glucose levels. Table 2 provides an overview of the seven different mini games and their learning objectives.

### 3.6. Virtual Coaching

The Virtual Coach component of the PERGAMON framework is designed to keep track of the personal objectives of each of the users as well as their achievements in the real world and in the game world. The coach uses this information to assist the users in achieving their objectives by processing collected data and offering guidance in the form of reminders, notifications and suggestions about certain actions and events.

**Figure 7:**
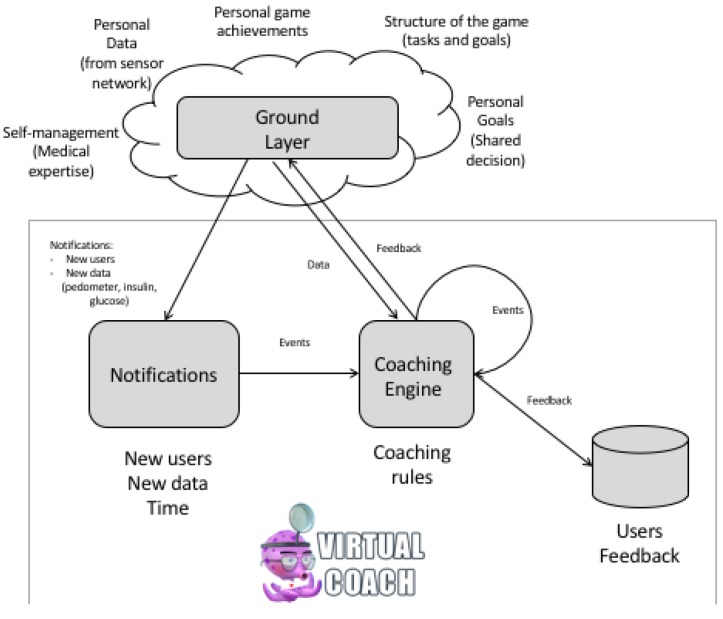
The coaching engine of the Virtual Coach of the PERGAMON platform depicting the knowledge and data source components used by the Virtual Coach.

**Figure 8:**
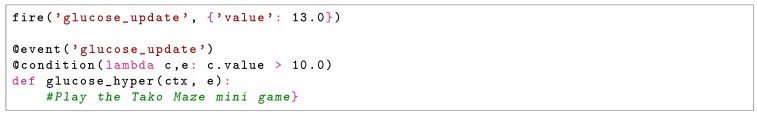
An example of coaching rule.

Figure 7 presents an overview of the virtual coach component. The data for the virtual coach comes from the Ground Layer. Data from the sensor network and data from the games can be accessed by the virtual coach component. Knowledge for the virtual coach is gathered from rules for self-management of T1D in cooperation with healthcare professionals. Personalization and tailoring of these self-management rules is done by defining personal goals between the client and the healthcare professionals (shared decision making). The Virtual Coach component consists of a coaching engine and a graphical representation of the virtual coach [16]. Coaching rules are defined in the coaching engine and allow for the analysis of data available in the Ground Layer derived from other components of the PERGAMON framework. The coaching engine is a rule- based engine based on the ECA (event, condition and action) engine developed at the University of Twente [16]. A typical coaching rule looks like the one in Figure 8. The rule (named ’glucose_hyper’ in this example) is executed based on an event (in this case the event ’glucose_update’), triggered by an event from the Ground Layer (in this case a trigger from the sensor network of a new measure of value 13.0). The rule is only executed when the value of the new measurement is greater than 10.0 (a blood glucose level of 10 or higher is called a hyperglycemia). Conditions can be general fixed values (such as hypoglycemia or hypoglycemia) or personals goals (such as the number of steps or the number of times a user wants to measure their blood glucose levels). The action of this coaching rule is a suggestion to play a mini game.

**Figure 9:**
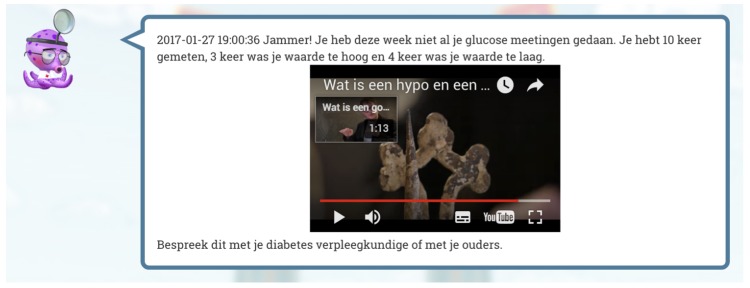
Example of a coaching message on the PERGAMON website. The message is in Dutch (English: *“Too bad! This week you did not do all your glucose measurements. You measured 10 times, 3 times your level was too high and 4 times your level was too low. Please, discuss this with your diabetes nurse or with your parents.”*).

The coaching rules are executed by triggers that are event- or time-based. Event based triggers are notifications from the sensor network or other components of the PERGAMON framework via the Ground Layer. Examples of triggers for coaching events are the creation of new users, data from sensors or achievements in the game(s). Time based triggers are based on the time of day and related to self-management (e.g., measuring glucose levels before dinner or before sleeping). All events are processed by the coaching engine and the result of these coaching rules can be a dialogue act of several kinds such as a *suggestion* (e.g., to play mini games where the user can meet educational objectives), a *reminder* or a *notification* (e.g., when personal goals are reached or users forget to do their measurements) or *hints* about how to continue in the main game. The Virtual Coach is able to send text messages and notifications to the website and to the Android application. By presenting the Virtual Coach as a cartoon-like character from the main game world, the coach connects the in-game world with the primary world of the user’s daily care activities. Figure 9 gives an example of a coaching message presented on the website including an educational movie with extra background information.

## 4. User Evaluations

After a two week pilot test with seven patients with T1D another group of 14 patients from the participating hospital in Ede tested the system over 6–8 weeks.

### 4.1. Goals

The user evaluation aimed to see how patients with T1D experience the game, a core element of gaming [57]. We used a mixed methods approach (i.e., direct observation, questionnaires, and semi structured interviews) to answer the following questions: Do players like to step into the “magic circle” [58] and does striving for progress in the game motivate healthy behaviours? Do users appreciate the coaching messages? Do users find the educational films useful? Do users find it difficult to use the system? Do users encounter any technical problems they cannot solve?

### 4.2. Materials and Methods

#### 4.2.1. Participants

Twenty-one adolescents with Type 1 diabetes participated in a pre-pilot (*n* = 7) and pilot study (*n* = 14). Participants were registered patients at the pediatric diabetes department from the Gelderse Vallei Hospital in Ede (the Netherlands). The first group of participants was allocated to a pre-pilot study in September 2016 and the second group of participants was allocated to a subsequent pilot study performed during October–December 2016. Participants were included if they fulfilled the following inclusion criteria: (a) diagnosis of Type 1 diabetes for at least six months; (b) between 12 and 18 years of age; (c) written informed consent provided by adolescents themselves and one of their parents (or legal guardians); (d) availability of an Android smartphone with an internet/data connection and (e) a reasonable level of understanding of the Dutch language. If the adolescent did not have an Android smartphone available, they were provided with a phone that satisfied system requirements.

#### 4.2.2. Design

The first group (*n* = 7) pre-pilot tested the PERGAMON platform for two weeks at home. Based on the results several suggestions were made and implemented to improve the platform. The second group (*n* = 14) pilot tested the adjusted PERGAMON platform for six to eight weeks at home. Both studies used a pre-post test design with a mixed-method approach in which questionnaires as well as open-ended interview questions were used.

#### 4.2.3. Procedure

Adolescents and their parents were contacted and informed about the study by their pediatrician or specialized diabetes nurses working at the pediatric diabetes department of Gelderse Vallei Hospital in Ede (the Netherlands) where both studies were performed. Adolescents who expressed their interest in participating in the study and fulfilled the inclusion criteria were sent an information letter including an informed consent form. After two weeks, participants were contacted to see if they had any further questions and to plan the first hospital visit. During the first hospital visit, participants were informed about the specific components of the PERGAMON platform (i.e., sensor network, virtual coach and serious game) in more detail and were offered support with downloading and installation of the applications on their Android device. The pre-pilot group participants evaluated the platform for two weeks at home and the subsequent pilot group participants were allowed to use the PERGAMON platform for six to eight weeks at home. No instructions were given for doing specific tasks. This was done to stimulate the report of technical or other issues naturally encountered during use in real life. A technical help desk, covered by the nurses and researchers involved, was available during the study period through contact by email or phone during office hours. Apart from the online questionnaires filled in during the pre- and post-test hospital visits, participants had the opportunity to share their experiences concerning usability and acceptability in a focus group during the second hospital visit.

The focus group was guided by a short outline presented through PowerPoint and ended with an open discussion about suggestions on how to improve the performance and adequacy of the platform. The focus group was moderated by a researcher experienced in usability research and testing. A second researcher (i.e., nurse) was present and made field notes.

All participants and one of their parents (or legal guardians) gave their informed consent for inclusion before they participated in the study. Both studies were conducted in accordance with the Declaration of Helsinki, and the protocol was approved by the Ethics Committee of the Gelderse Vallei Hospital (BC/1606-383) on 1st of August 2016 and by Coventry University (P45142) on 29th of June 2016.

#### 4.2.4. Measures

Socio-demographic questionnaire—A self-constructed socio-demographic questionnaire was used to collect socio-demographic information (e.g., gender, age, ethnic background) and general information concerning participants’ previous smartphone use.

Problem Areas in Diabetes Questionnaire [59]—The Dutch translation of the Problem areas in Diabetes (PAID) was used to examine diabetes related emotional distress. It consists of 20 items on a 5-point Likert scale. An example item is “Feeling scared when you think about living with diabetes”. In the current study, the total PAID score was used as an indication of the patient’s diabetes related emotional distress before and after using the PERGAMON platform. A score of 40 or higher is considered to be an indication of “emotional burnout”. This questionnaire demonstrates good psychometric characteristics and the Dutch version used has been validated with patients in the Netherlands [59].

System Usability Scale (SUS; Dutch translation [60])—The Dutch translation of the SUS was used to measure usability of the PERGAMON platform. This questionnaire consists of 10 items on a 5-point Likert scale ranging from strongly agree to strongly disagree. An example item is “I thought the system was easy to use”. A score above 68 is interpreted as above average usability and below 68 is interpreted as below average. This questionnaire is internationally acknowledged as a reliable questionnaire to measure system usability [60].

Semi-structured interview—A semi-structured interview was used during group discussions (focus groups) to explore user experiences of the PERGAMON platform with regard to design, enjoyment, storyline, ease of use, educational value, engagement and innovative aspects of the platform (e.g., the virtual coach, sensors, serious games), as well as their overall judgment about the PERGAMON platform.

## 5. Results

Background characteristics and smartphone use of both study groups are presented in Table 3. We asked participants “Do you expect that the PERGAMON platform will support you in your diabetes management?”. Of the 21 participants in the pre-pilot and the pilot groups 7 (33.33%) answered positively; 1 (4.76%) answered negatively, and 13 (61.90%) said they did not know.

In total, 71 adolescents with T1D were approached to take part in the pilot study, of which 30 patients met the inclusion criteria and indicated their interest to participate in the pilot study. Sixteen participants (7 males/9 females) withdrew their consent because of delayed study start date (13%), time constraints due to school activities (56%), time constraints due to activities outside school hours (13%), low motivation to participate (6%), low expectations regarding the game format (6%) and worries about their diabetes condition (6%).

During the pre-pilot study there was one drop-out during the second hospital visit due to academic under achievement at school.

### 5.1. Problem Areas in Diabetes Questionnaire

Participants in the pre-pilot study demonstrated a mean PAID score of 21.25 (SD = 21.21; range = 0.00–55.00) at the first hospital visit and a mean PAID score of 10.63 (SD = 8.62; range = 0.00–22.50) at the second hospital visit after two weeks. Furthermore, participants in the pilot study showed a mean PAID score of 16.52 (SD = 14.17; range = 0.00–52.50) at the first hospital visit and a mean PAID score of 12.95 (SD = 9.84; range = 0.00–31.25) at the second hospital visit. Results indicate that participants did not experience a great deal of diabetes related emotional distress (score < 40) at baseline or after using the PERGAMON platform.

### 5.2. System Usability Scale

After using the PERGAMON platform, participants from the pre-pilot study group demonstrated a mean SUS index of 44.58 (SD = 21.18; range = 25.00–82.50) which indicates a below average usability score. Almost all participants indicated that they found the PERGAMON platform unnecessarily complex (statement 2) and not easy to use (statement 3). Half of the participants felt that they needed assistance to be able to use the PERGAMON platform (statement 4; Table 1). In the pilot-study, group participants had a mean SUS index of 50.18 (SD = 13.71; range = 35.00–90.00) which indicates a below average usability score. As can be seen in Table 4, although 50% of pilot study participants thought the PERGAMON platform was easy to use after playing it for two weeks (statement 3) and 57% did NOT think (disagreed) they would need assistance when using the system (statement 4); 57% did NOT think (disagreed) that they would like to use the system frequently (statement 1).

### 5.3. Semi-Structured Interview

PERGAMON platform—One participant in the pre-pilot study group indicated that he never used the platform. Three participants (43%) indicated that they used the PERGAMON platform a couple of times a week and the remaining participants said that they used the platform (almost) every day. The average usage time was 10 h (ranging from 10 min to 40 h). Four participants from the pilot study group indicated that they never used the platform. However, most of the participants (57%) indicated that they used the PERGAMON platform once a week and two participants used the platform more often. The time that they used the platform ranged between 10 min and 18 h. On average, participants from the pre-pilot group rated the game (“How would you rate the game on a scale of 1 to 10?”) with a 6.83 (SD = 0.98; on a 10-point scale), whereas the pilot study group participants rated the game with a 6.07 (SD = 1.44). Overall, most participants (except for five) in both study groups encountered technical problems such as failures in transfer of blood glucose values to the PERGAMON platform, or low performance of the game. In total, only four participants felt the PERGAMON platform supported them in their diabetes management. Suggestions to improve the platform include making the game less complex/difficult to play, change the appearance of the game to fit with its target group and better communication between the different components of the platform. Most participants appreciated that the game set real-world goals.

TikiTako adventure game—Three participants (43%) indicated that they played the game a couple of times a week and the remaining participants indicated they played the TikiTako adventure game (almost) every day. The average playtime was 8 h (ranging from 15.00 min to 40.00 h). Four participants (29%) indicated that they played once a week and another four participants (29%) indicated they played once a month. One participant played the adventure game almost every day. The time that they played the game ranged between 15 min and 9 h. On average, participants from the pre-pilot group rated the game with a 5.67 (SD = 2.34; on a 10-point scale), whereas the pilot study group participants rated the game with a 6.07 (SD = 1.98). Both groups rated the game as difficult to play (>8.00 on a 10-point scale) as there was not enough explanation given or explanations were too cryptic. Participants from the pre-pilot study group scored 8.50 on average (SD = 1.38) whereas participants from the pilot study scored 8.43 on average (SD = 0.65). In total, four participants (19%) in both study groups indicated that they learned from the game, namely refreshing diabetes knowledge. Also more than half of the participants in the pre-pilot group (57%) and in the pilot group (50%) want to get access to this game once it has been fully developed. Moreover, a large part of the pre-pilot group (57%) and of the pilot group (64%) would recommend the game to other participants with T1D, because “you can acquire diabetes knowledge” and “because you become more motivated concerning self-management”. Suggestions to improve the game include the appearance, the look-and-feel, the solving of technical issues, more interesting mini games and making it less difficult to finish levels.

Mini games—Overall, participants rated the seven mini games for likability in a range from 4.42 (SD = 2.78) to 7.33 (SD = 2.52; on a 10-point scale) with Tako Maze indicated as the most popular mini game in the pre-pilot study group and Ramen Master as the most popular in the pilot study group. Participants from the pre-pilot study group indicated that the mini game could be improved by offering more explanation for certain mini games or that the difficulty level should be adaptive and set by the user themselves. Furthermore, participants from the pilot study suggested that there should be a better technical performance of the mini games (e.g., response time to tapping the screen) and a more mature look-and-feel of the game.

Virtual coach—All participants in the pre-pilot study (except for one) indicated that they received messages from the virtual coach, ranging from 3 to 8 messages. Almost all participants (except for one) understood the virtual coach messages that were mainly focused on blood glucose measurement. Participants indicated on a 7-point Likert scale to which degree the coach was up-to-date (mean = 4.67; SD = 2.58), supportive (mean = 3.50; SD = 2.07), friendly (mean = 5.33; SD = 1.63) and pleasant (mean = 4.67; SD = 1.21). Almost all pilot study participants (except for three) indicated that they received messages from the virtual coach, ranging from 2 to 10 messages. All participants understood the messages of the virtual coach and indicated on a 7-point scale that the coach was up-to-date (mean = 4.21; SD = 1.72), supportive (mean = 3.79; SD = 1.81), friendly (mean = 5.07; SD = 1.39) and pleasant (mean = 4.50; SD = 1.79). According to the participants in the pre-pilot study, the virtual coach could be improved by adding the capability of answering their questions and by appearing less often on their phone screen as an overlay. Participants in the pilot study group suggested that the virtual coach could be improved by decreasing the frequency of messages and by offering the user to turn the virtual coach’s messages on or off.

### 5.4. Focus Groups

TikiTako adventure game—With regard to the adventure game, participants from the pre-pilot study indicated that they got stuck after a few levels. It was too difficult for them to continue the game and therefore they stopped playing. Several participants said that the hints in the game were too cryptic and that advice from the virtual coach (as a game guide) was difficult to understand. Furthermore, they said the look and feel of the game was childish and did not match the difficulty level of the game. Even after in-game feedback was simplified and made less cryptic, participants from the pilot study generally indicated that the TikiTako adventure game was too childish in terms of its graphics and storyline and this simplicity did not match with the difficult and highly complex puzzles.

Mini games—Participants from both study groups indicated that the interface was not user friendly as users did not know they could use the “help” button to get more information about how to play the game. One participant suggested that it might be good to receive information about this before or during the mini game. In addition, participants found the (menu) buttons too small. Mini games about food and intake of insulin were seen as more useful for children recently diagnosed with diabetes. Furthermore, the look and feel of the mini games was described as childish and did not match with the difficulty level of the mini games. Even after the number of points needed to reach the goal at the end of the games was lowered, puzzles remained highly complex according to the pilot study participants.

Virtual coach—Participants in both study groups were positive about the timing of messages and the number of reminders sent. Participants also provided positive feedback about the weekly overview messages given by the virtual coach, although they were not noticed by all participants. One participant from the pre-pilot study explained that receiving messages (or notifications) from the virtual coach helped to support their self-management. Participants in the pre-pilot group indicated that an overlay image of the virtual coach on their screen was annoying when they received a message from the coach. Therefore, the interface of the virtual coach was revised for the pilot study (i.e., messages were made clickable so that the whole message was visible at first instance) so that participants could be aware that more content was available than just the label text on the message box. Participants in both the pre-pilot and the pilot study said that educational movies presented by the virtual coach about diabetes were nice but became boring after multiple viewings. During both studies participants said that because of synchronization problems and requirements to enter data on a daily basis (and not after each measurement), feedback from the virtual coach was not up-to-date and therefore perceived as incorrect. More importantly, the coach was not developed to respond immediately to high or low glucose readings leading participants to suggest that it would be good if the virtual coach could send messages when measurements differed from normal values.

Sensors—Participants from both study groups liked the idea that sensor data can automatically be sent to the PERGAMON platform. However, not every participant was enthusiastic about wearing a Mi-Fit band or changing their trusted blood glucose meter for a new meter that automatically sends the glucose data to the platform (i.e., most participants used a pump and other glucose monitors). Entering the glucose measurements by hand was perceived as a lot of extra work and a hurdle with this technology.

## 6. Discussion

This report presented a proof of concept of a pervasive gamified platform framework that is a unique combination of integrated sensors (wearables), a virtual coach and serious games in healthcare. This description of this platform contributes to research on pervasive technology approaches in healthcare [49,61]. Behaviour change theories and game design principles underpinning the framework were presented with particular attention to how these theories and principles were combined and applied to develop content across the technology components to promote positive health behaviours among young people with T1D. This work contributes to efforts to link theories more closely to interventions in the research literature [62]. This report also presented the results of pilot tests of early prototypes of the framework. Consistent with previous literature documenting issues that often arise in early evaluations of prototypes, [18,61,63,64], we found that similar issues of usability and technical quality assurance presented challenges for users. We received several comments that the games were difficult to play and were “overly complex” due to design issues and should be more simple and straightforward to play. At the same time, participants commented that the “look and feel” of the games seem to be targeted to a much younger audience and should be designed for a more mature audience. These issues made it difficult to answer some of the original questions of the research (e.g., Do players like to step into the “magic circle”, and Does striving for progress in the game motivate healthy behaviours). In addition, we were unable to evaluate fundamental questions about use of the platform and engagement in health behaviours because our findings did not confirm a widespread assumption in the literature that users carry their mobile platforms with them “everywhere” and that the platforms are constantly connected to data networks [61,63,65]. Specifically, we found that because of the central server architecture, progress in the game and coaching critically depended on the user being continuously connected. What became clear from the evaluation “in the wild” is that we incorrectly assumed that users would be continuously online, they would wear their physical activity wrist band all day and they would consistently send their BG data to the sensor network immediately after measuring. Some carried their own mobile phone (not the phone provided) and used the phone provided for gaming only at home. This led to a less than optimal user experience. Thus, the prototype we evaluated was not truly “pervasive” in practice given technology constraints and individual differences in user preferences for using the technology. Finally, the variation in use and feedback of the platform also supports claims that simply applying gamification principles to an intervention does not guarantee increased engagement with a technology, much less, increased motivation to engage in health behaviours targeted by the intervention [49]. The games, and the platform overall, could be improved by simplifying the interface so that it is more intuitive and easy to use, reducing the difficulty of the games for increased playability, modifying the look of the game so that it is more appealing to the target users, and addressing technical issues reported so that the platform is free of major technical bugs that inhibit enjoyment and prevent use of the technology. The implementations of these improvements should be further evaluated because recent research suggests that participatory design processes do not always lead to positive effects on health behaviours [66].

The results from the pilot study contribute to and further the literature on challenges faced when deploying pervasive technologies in healthcare [61]. The negative feedback we report in particular is significant because it presents a more balanced report of technologies (reporting negative as well as positive findings) that contribute to theoretical and practical progress in the field. This addresses a pervasive problem that negative reports of findings including negative user feedback of new technologies are not reported [23,61,67]. The findings from the pilot study also contribute to research on the state of information technologies that suggest that more reliable technology systems should be developed and deployed in healthcare [68]. In sum, the balanced reviews provided in this report not only contribute to scientific efforts to understand the application of technology to healthcare but may also be of practical value for informing the strategic planning of technology development efforts to improve healthcare. Specifically for the latter, in the future, evaluations of similar prototypes such as ours should consider the current findings that suggest specific areas of challenge (e.g., usability, acceptability of look and feel, technical performance) should be addressed before evaluating the impact of the prototype on health outcomes.

The pilot studies show the importance of finding a good balance between flawless and non-obtrusive technology, between challenging but not too difficult game play, and the relevance of designing and fine-tuning to the age group as well as to the level of knowledge and experience of the individual user. Furthermore, the use of mobile technology in combination with web-based elements has been shown to be feasible with potential to change healthcare by supporting a vulnerable group of patients in self-managing their chronic disease. We emphasise the value of mixed methods long-term user studies “in the wild” with patients from the target group in their daily environment, as well as the involvement of the medical team in their role as care providers in these types of user studies.

### 6.1. Limitations

The current study demonstrates the design and evaluation of a pervasive coaching and gamification platform within a clinical sample of young diabetes patients. However, the findings in this report are limited. The platform evaluated in this research was of a prototype version of the framework. As such, difficulties with usability and seamless functioning of the technology were challenges in evaluating basic research questions regarding user engagement and impact on outcomes. In terms of research design, the small sample size of participants and relatively high attrition rate, seen in similar studies of smartphone apps for health (e.g., [69]), suggest that the results may have limited generalizability to the broad population of potential users. Also, because users of prototypes in our studies were not compared to a control group of users of comparable prototypes, it is not clear how much their feedback was specific to the content and design of the prototypes we tested, or could be attributed to general issues common with early versions of technologies.

### 6.2. Future Research

Future research efforts to develop complex and integrative platforms such as the platform described in this study should incorporate user input and co-design approaches and rigorous quality assurance testing before being subjected to user evaluation studies. The ideal of a user-centered design process, common to the field of human-computer interaction, that engages target users early and often [46] is sometimes hard to realize but considered necessary for successful design. Future studies should also address issues of deployment by engaging with key stakeholders in healthcare in addition to patients as end-users to facilitate the ultimate success of the technology in “real world” settings. In terms of methodology, future research on platforms such as the PERGAMON platform in this study, should be conducted with larger groups of patients/users (including those recently diagnosed) with rigorous research designs and in a hospital setting over a longer period to see if indeed gamification helps patients to automate self-care behaviours. If proven effective, further studies should aim to examine and clarify which features and game elements contribute to a positive user experience and increase self-care for patients with a chronic condition.

### 6.3. Implementation Challenges of Serious Gaming and Virtual Coaching in Healthcare

There are various challenges on the way to the implementation of a pervasive integrated system such as the PERGAMON gamification platform into the practice of hospitals in their care for chronic diseases. It is essential that the technology used to enhance self-management, is integrated in the daily routine of the patients in a non-obtrusive way [10,11,12]. Every step that requires extra actions on top of all obligatory procedures for the user has to be avoided. During the past years some progress has been made in simplifying blood glucose monitoring [5], but these systems are expensive and not-reimbursed. Simplification, unobtrusiveness, safety, reliablity and robustness are prerequisites for introducing new techniques such as the PERGAMON platform.

Prejudice and stigmatization play important roles in the acceptance of tools and devices that support disease management [70,71]. This is not only the case for medical devices, but also when the game, or platform components like an activity tracker, or a sensor look clumsy or childish, they may become objects of mockery [72]. We learned from the user evaluations how essential a good balance is between flawless and non-obtrusive technology, challenging but not to difficult gameplay, and excellent fine-tuning to the age group.

In health care a rather conservative attitude often exists toward new developments. An important reason is that, because of safety precautions, proven technology is preferred above new techniques. So far, proof of the effectiveness of serious gaming is considered insufficient, and privacy issues or cyber-crime are increasingly considered to be a risk. Furthermore, the ICT departments in health care institutions are fully focused on medical systems and do not have the knowledge, the experience or the willingness to deal with other applications before their added value is demonstrated. Research has shown that the perception of facilitators, i.e., the degree to which an individual believes that an organizational and technical infrastructure exists to support the use of the system, is one of the most important variables to consider for increasing doctors’ and nurses’ intention to use the new technology for telemedicine [73].

A second factor that impedes the introduction of complex digital platforms may be a financial one. Hospitals and other healthcare systems are non-profit organisations and the return on investments for serious gaming and virtual coaching is still very uncertain. Patients on the other hand are used to the fact that the costs of healthcare are largely reimbursed by health insurance companies. Before they are willing to pay for the apps and wearables, they have to be convinced that playing, learning and being coached by a gaming platform offers them great benefits. Health insurance companies will only reimburse the costs of these platforms when there is proof of savings or an explicit and measurable increase in the quality of health care, which is hardly ever the case. Finally, many patients with chronic diseases have long-standing habits of performing their repetitive actions in a certain way and with a particular device. Technology acceptance studies in health care showed that changing to new devices (e.g., blood glucose meters) will only be accepted by the patients if the benefits that they experience are very clear [73].

## 7. Conclusions

This paper provided an overview of an innovative integrated pervasive gamified platform that combined sensors, mobile technologies, virtual coaching and serious games. The description of the platform provided insight into how theory could be applied to the design of behaviour change technology in healthcare. The findings from the user study contributed to knowledge about challenges faced when developing and testing complex behaviour change technologies in healthcare. They have practical significance for strategic planning for successful technology development of behaviour change interventions in healthcare.

## Figures and Tables

**Table 1:** Main characteristics and game elements of the *Tako Game* for diabetic children (see Figure 6).

1	Theme: in the world of the Takos, an evil monk has stolen the sacred tentacle from the Temple. T-Shan is called in by the Great Red Tako to retrieve it. In order to accomplish his mission, T-Shan must overcome a series of challenges, helped by the player (the patient), who can guide T-Shan and obtain precious suggestions and points in the Real World.
2	In order to gain points to help T-Shan in the world of the Takos, the player had to accomplish the empowerment goals in his/her game dashboard. The empowerment goals were related to the following activities: Monitoring blood glucose levels (diary entries, use of a sensor device such as glucometer) Acquiring skills and knowledge (learn carbohydrate counting, how to balance a healthy meal, etc.) Social activities (set up a personal profile, use the wall) Other goals are also directly linked to the game itself e.g., requiring the player to play a mini game to be able to proceed to another scene in the temple. Players are never alone in their quest: they are helped by the Great Red Tako (i.e., the virtual coach), who supports and provides motivational messages to help them in accomplishing the set goals.
3	Each therapeutic goal is made up of several tasks that are rewarded with points when completed. Some can be carried out online (e.g., take an “educational candy”, i.e., view a learning resource, watch a video, read a document; or “fill in the diary”); others may be carried out by the user in their daily activities and tracked thanks to wearable sensor devices (glucometer and pedometer) that automatically detect and synchronise data about blood glucose level and physical activity, so that the PERGAMON system knows that a task has been carried out.
4	Points are of three types and each type is linked to a specific type of action: Experience: gained through the game to contribute to the player’s ranking in the general leaderboard Knowledge: used in the world of Tako to unlock some locations Evolution: used to make the Tako avatar grow and acquire graphical assets
5	Points are used to get suggestions (and thus solve a riddle or overcome an obstacle), or get an object that can be used later on to do something in the game in the world of the Takos.
6	The virtual coach, situated in the real world, can affect the game world of the Takos by providing suggestions on some tasks particularly helpful for the users that can help them gain points advising players to complete a goal related to the levels in the game.

**Table 2:** Overview of the mini games in the *Tiki Tako Game*. In the left colom the name of the game and a screen capture, on the right side the learning objective related to this mini game.

Mini Game		Learning Objective
Ramen Master	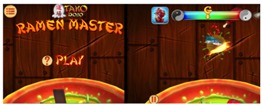	The objective is to teach and test the knowledge of the player about the types of food to eat according to the blood glucose levels.
Tako Maze	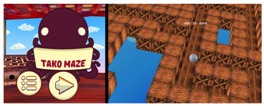	The objective of the game is to teach and test the knowledge of the player about the carbohydrates amount present in different food items.
Tako Doctor	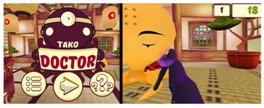	To reinforce the best practice around the stages necessary in administering an insulin injection. Players will solve a simple drawing puzzle alongside each key step of the injection process, with the theory that the repetition of playing the game will begin to ingrain the steps in memory.
Tako Chef	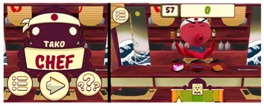	Tako Chef aims to increase awareness of how certain foods and diabetic items affect blood glucose levels. The player must learn to quickly identify an item that is suitable (among unsuitable items) for a given blood glucose level.
Tako Runner	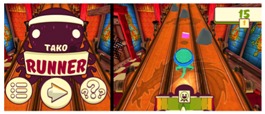	Tako Runner aims to help the player understand how exercise and diet affect blood glucose levels, and that exercise should stop when those levels are too low or high.
Tako Swimmer	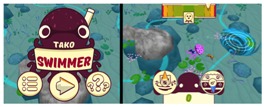	Tako Swimmer aims to help the player understand how exercise and diet affect blood glucose levels, and that exercise should stop when those levels are too low or high.
Tako Explorer	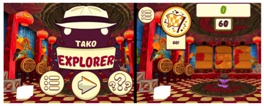	Tako Explorer aims to familiarize the player with key terminology used when talking about diabetes.

**Table 3:** Socio-demographic characteristics and smartphone use in both study groups (*N* = 21).

	Total (*N* = 21)	Pre-pilot (*n* = 7)	Pilot (*n* = 14)
Male	10 (48%)	2 (29%)	8 (57%)
Female	11 (52%)	5 (71%)	6 (43%)
Age (years), mean	13.90	14	13.86
Smartphone use in general? *n* (%)			
Social media	3 (14.29%)	1 (14.29%)	2 (14.29%)
Search information	2 (9.52%)	2 (28.57%)	0 (0%)
Call/Text/Whatsapp	8 (38.09%)	3 (42.85%)	5 (35.7%)
Listen to music	5 (23.81%)	0 (0%)	5 (35.7%)
Other	3 (14.29%)	1 (14.29%)	2 (14.29%)
Smartphone use for health? *n* (%)			
Monitoring physical activity	2 (9.52%)	1 (14.29%)	1 (7.14%)
Monitoring nutrition	1 (4.76%)	1 (14.29%)	0 (0%)
Monitoring blood glucose	1 (4.76%)	0 (0%)	1 (7.14%)
No monitoring	17 (80.95%)	5 (71.42%)	12 (85.72%)
Favourite computer genre? *n* (%)			
Puzzle	2 (9.52%)	1 (14.29%)	1 (7.14%)
Action	8 (38.09%)	4 (57.14%)	4 (28.57%)
Racing	1 (4.76%)	0 (0%)	1 (7.14%)
Multi-player	4 (19.04%)	0 (0%)	4 (28.57%)
Other	6 (28.57%)	2 (28.57%)	4 (28.57%)

**Table 4:** Pre-pilot and pilot study usability (SUS) questionnaire scores (in %).

	Disagree (1–2)		Neutral (3)		Agree (4–5)	
	Pre	Pilot	Pre	Pilot	Pre	Pilot
*Positive items*						
1: I think that I would like to use this system frequently	50.0	57.1	33.3	21.4	16.7	21.4
3: I thought the system was easy to use	66.7	28.6	33.3	21.4	0	50.0
5: I found the various functions in this system were well integrated,	33.3	50.0	33.3	35.7	33.3	14.3
7: I would imagine that most people would learn to use this system very quickly	50.0	57.1	16.7	14.3	33.3	28.6
9: I felt very confident using the system	33.3	42.9	50.0	35.7	16.7	21.4
*Negative items*						
2: I found the system unnecessarily complex	16.7	35.7	0	28.6	83.3	35.7
4: I think that I would need assistance to be able to use this system	50.0	57.1	16.7	28.6	33.3	14.3
6: I thought there was too much inconsistency in this system	33.3	50.0	33.3	28.6	33.3	21.4
8: I found this system very cumbersome/awkward to use	33.3	42.9	33.3	35.7	33.3	21.4
10: I needed to learn a lot of things before I could get going with this system	33.3	50.0	0	28.6	66.7	21.4

## References

[1] Grady P.A., Gough L.L. (2014). Self-Management: A Comprehensive Approach to Management of Chronic Conditions. Am. J. Public Health.

[2] Diaz-Valencia P.A., Bougnères P., Valleron A.J. (2015). Global epidemiology of type 1 diabetes in young adults and adults: A systematic review. BMC Public Health.

[3] Mauras N., Fox L., Englert K., Beck R.W. (2013). Continuous glucose monitoring in type 1 diabetes. Endocrine.

[4] Hood K.K., Peterson C.M., Rohan J.M., Drotar D. (2009). Association Between Adherence and Glycemic Control in Pediatric Type 1 Diabetes: A Meta-analysis. Pediatrics.

[5] Markowitz J.T., Harrington K.R., Laffel L.M.B. (2013). Technology to Optimize Pediatric Diabetes Management and Outcomes. Curr. Diabetes Rep..

[6] Van der Burg G. mHealth in diabetes management—The BLink experience. Proceedings of the 39th ISPAD Annual Conference 2013.

[7] Patton S.R. (2015). Adherence to Glycemic Monitoring in Diabetes. J. Diabetes Sci. Technol..

[8] Weinzimer S.A., Beck R.W., Chase H.P., Fox L.A., Buckingham B.A., Tamborlane W.V., Kollman C., Coffey J., Xing D., Ruedy K.J. (2005). Accuracy of Newer Generation Home Blood Glucose Meters in a Diabetes Research in Children Network (DirecNet) Inpatient Exercise Study. Diabetes Technol. Ther..

[9] Holl R., Swift P., Mortensen H., Lynggaard H., Hougaard P., Aanstoot H., Chiarelli F., Daneman D., Danne T., Dorchy H. (2003). Insulin injection regimens and metabolic control in an international survey of adolescents with type 1 diabetes over 3 years: Results from the Hvidore study group. Eur. J. Pediatr..

[10] Rausch J.R., Hood K.K., Delamater A., Pendley J.S., Rohan J.M., Reeves G., Dolan L., Drotar D. (2012). Changes in treatment adherence and glycemic control during the transition to adolescence in type 1 diabetes. Diabetes Care.

[11] Pyatak E.A., Florindez D., Weigensberg M.J. (2013). Adherence decision making in the everyday lives of emerging adults with type 1 diabetes. Patient Preference Adherence.

[12] Holtz B.E., Murray K.M., Hershey D.D., Dunneback J.K., Cotten S.R., Holmstrom A.J., Vyas A., Kaiser M.K., Wood M.A. (2017). Developing a Patient-Centered mHealth App: A Tool for Adolescents With Type 1 Diabetes and Their Parents. JMIR mHealth uHealth.

[13] Sabete E. (2003). Adherence to Long-Term Therapies: Evidence for Action.

[14] Goyal S., Nunn C.A., Rotondi M., Couperthwaite A.B., Reiser S., Simone A., Katzman D.K., Cafazzo J.A., Palmert M.R. (2017). A Mobile App for the Self-Management of Type 1 Diabetes Among Adolescents: A Randomized Controlled Trial. JMIR mHealth uHealth.

[15] Osborn C.Y., Mayberry L.S., Mulvaney S.A., Hess R. (2010). Patient Web Portals to Improve Diabetes Outcomes: A Systematic Review. Curr. Diabetes Rep..

[16] Op den Akker H., Klaassen R., Nijholt A. (2016). Virtual coaches for healthy lifestyle. Toward Robotic Socially Believable Behaving Systems-Volume II.

[17] Ye X. (2015). GlucOnline Coach: A virtual coach app for diabetes patients. Master’s Thesis.

[18] Brzan P.P., Rotman E., Pajnkihar M., Klanjsek P. (2016). Mobile Applications for Control and Self Management of Diabetes: A Systematic Review. J. Med. Syst..

[19] Chomutare T., Fernandez-Luque L., Arsand E., Hartvigsen G. (2011). Features of Mobile Diabetes Applications: Review of the Literature and Analysis of Current Applications Compared Against Evidence-Based Guidelines. J. Med. Internet Res..

[20] Eng D.S., Lee J.M. (2013). The Promise and Peril of Mobile Health Applications for Diabetes and Endocrinology. Pediatr. Diabetes.

[21] Boyle L., Grainger R., Hall R.M., Krebs J.D. (2017). Use of and Beliefs About Mobile Phone Apps for Diabetes Self-Management: Surveys of People in a Hospital Diabetes Clinic and Diabetes Health Professionals in New Zealand. JMIR mHealth and uHealth.

[22] Williams G.C., Freedman Z.R., Deci E.L. (1998). Supporting Autonomy to Motivate Patients With Diabetes for Glucose Control. Diabetes Care.

[23] Kato P. (2010). Video games in health care: Closing the gap. Rev. Gen. Psychol..

[24] Deterding S., Dixon D., Khaled R., Nacke L. From Game Design Elements to Gamefulness: Defining “Gamification”. Proceedings of the 15th International Academic MindTrek Conference: Envisioning Future Media Environments.

[25] Baranowski T., Buday R., Thompson D., Baranowski J. (2008). Playing for real: Video games and stories for health-related behavior change. Am. J. Prev. Med..

[26] Thompson D., Baranowski T., Buday R., Baranowski J., Thompson V., Jago R., Griffith M.J. (2010). Serious Video Games for Health How Behavioral Science Guided the Development of a Serious Video Game. Simul. Gaming.

[27] Cugelman B. (2013). Gamification: What It Is and Why It Matters to Digital Health Behavior Change Developers. JMIR Serious Games.

[28] Järvinen A. (2008). Games without Frontiers: Methods for Game Studies and Design. Ph.D. Thesis.

[29] Schell J. (2008). The Art of Game Design: A book of Lenses.

[30] Chomutare T., Johansen S., Arsand E., Hartvigsen G. (2016). Play and learn: Developing a social game for children with diabetes. Stud. Health Technol. Inform..

[31] Lieberman D. (2012). Video games for diabetes self-management: Examples and design strategies. J. Diabetes Sci. Technol..

[32] Aoki N., Ohta S., Masuda H., Naito T., Sawai T., Nishida K., Okada T., Oishi M., Iwasawa Y., Toyomasu K. (2004). Edutainment tools for initial education of type-1 diabetes mellitus: Initial diabetes education with fun. Medinfo.

[33] Aoki N., Ohta S., Okada T., Oishi M., Fukui T. (2005). INSULOT A cellular phone-based edutainment learning tool for children with type 1 diabetes. Diabetes Care.

[34] Pouw I.H. (2015). You are what you eat: Serious Gaming for Type 1 Diabetic Persons. Master’s Thesis.

[35] Brown S.J., Lieberman D.A., Gemeny B.A., Fan Y.C., Wilson D.M., Pasta D.J. (1997). Educational video game for juvenile diabetes: Results of a controlled trial. Med. Inform..

[36] Fuchslocher A., Niesenhaus J., Krämer N. (2011). Serious games for health: An empirical study of the game *Balance* for teenagers with diabetes mellitus. Entertain. Comput..

[37] Lieberman D.A. (2001). Management of chronic pediatric diseases with interactive health games: Theory and research findings. J. Ambul. Care Manag..

[38] Theng Y.L., Lee J.W., Patinadan P.V., Foo S.S. (2015). The Use of Videogames, Gamification, and Virtual Environments in the Self-Management of Diabetes: A Systematic Review of Evidence. Games Health J..

[39] Charlier N., Zupancic N., Fieuws S., Denhaerynck K., Zaman B., Moons P. (2015). Serious games for improving knowledge and self-management in young people with chronic conditions: A systematic review and meta-analysis. J. Am. Med. Inform. Assoc..

[40] Johnson D., Deterding S., Kuhn K.A., Staneva A., Stoyanov S., Hides L. (2016). Gamification for health and wellbeing: A systematic review of the literature. Internet Interv..

[41] Deacon A.J., O’Farrell K. The use of serious games and gamified design to improve health outcomes in adolescents with chronic disease: A review of recent literature. Proceedings of the International Conference on Successes and Failures in Telehealth.

[42] Sardi L., Idri A., Fernández-Alemán J.L. (2017). A systematic review of gamification in e-Health. J. Biomed. Inform..

[43] Kumar V.S., Wentzell K.J., Mikkelsen T., Pentland A., Laffel L.M. (2004). The DAILY (Daily Automated Intensive Log for Youth) trial: A wireless, portable system to improve adherence and glycemic control in youth with diabetes. Diabetes Technol. Ther..

[44] Cafazzo J.A., Casselman M., Hamming N., Katzman D.K., Palmert M.R. (2012). Design of an mHealth App for the Self-management of Adolescent Type 1 Diabetes: A Pilot Study. J. Med. Internet Res..

[45] Klingensmith G.J., Aisenberg J., Kaufman F., Halvorson M., Cruz E., Riordan M.E., Varma C., Pardo S., Viggiani M.T., Wallace J.F. (2013). Evaluation of a combined blood glucose monitoring and gaming system (Didget) for motivation in children, adolescents, and young adults with type 1 diabetes. Pediatr. Diabetes.

[46] Klasnja P., Consolvo S., McDonald D.W., Landay J.A., Pratt W. Using mobile & personal sensing technologies to support health behavior change in everyday life: Lessons learned. Proceedings of the American Medical Informatics Association (AMIA) Annual Symposium.

[47] Lauritzen J., Årsand E., Horsch A., Fernandez-Luque L., Chomutare T., Bellika J., Hejlesen O., Hartvigsen G. Social media and games as self-management tools for children and adolescents with type 1 diabetes mellitus. Proceedings of the International Conference on Health Informatics, HEALTHINF 2012.

[48] McCarthy G.M., Rodríguez Ramírez E.R., Robinson B.J., de Vries P.W., Oinas-Kukkonen H., Siemons L., Beerlage-de Jong N., van Gemert-Pijnen L. (2017). Letters to Medical Devices: A Case Study on the Medical Device User Requirements of Female Adolescents and Young Adults with Type 1 Diabetes. Persuasive Technology: Development and Implementation of Personalized Technologies to Change Attitudes and Behaviors, Proceedings of the 12th International Conference, PERSUASIVE 2017, Amsterdam, The Netherlands, 4–6 April 2017.

[49] Baranowski T., Blumberg F., Buday R., DeSmet A., Fiellin L.E., Green C.S., Kato P.M., Lu A.S., Maloney A.E., Mellecker R. (2016). Games for Health for Children—Current Status and Needed Research. Games Health J. Res. Dev. Clin. Appl..

[50] Van Wynsberghe A. (2012). Designing Robots with Care. Ph.D. Thesis.

[51] Burke S.D., Sherr D., Lipman R.D. (2014). Partnering with diabetes educators to improve patient outcomes. Diabetes Metabol. Syndr. Obes. Targets Ther..

[52] Oinas-Kukkonen H., Harjumaa M. (2009). Persuasive Systems Design: Key Issues, Process Model, and System Features. Commun. Assoc. Inf. Syst..

[53] Abraham C., Michie S. (2008). A taxonomy of behavior change techniques used in interventions. Health Psychol..

[54] Locke E.A., Latham G.P. (2002). Building a practically useful theory of goal setting and task motivation: A 35-year odyssey. Am. Psychol..

[55] Patrick H., Williams G.C. (2012). Self-determination theory: Its application to health behavior and complementarity with motivational interviewing. Int. J. Behav. Nutr. Phys. Act..

[56] Oinas-Kukkonen H., Ploug T., Hasle P., Oinas-Kukkonen H. (2010). Behavior Change Support Systems: A Research Model and Agenda. Persuasive Technology.

[57] Calvillo-Gámez E.H., Cairns P., Cox A.L. (2010). Assessing the core elements of the gaming experience. Evaluating User Experience in Games.

[58] Huizinga J. (1955). Homo Ludens: A Study of the Play-Element in Culture.

[59] Snoek F.J., Pouwer F., Welch G.W., Polonsky W.H. (2000). Diabetes-related emotional distress in Dutch and US diabetic patients: Cross-cultural validity of the problem areas in diabetes scale. Diabetes Care.

[60] Brooke J. (1996). SUS-A quick and dirty usability scale. Usabil. Eval. Ind..

[61] Orwat C., Graefe A., Faulwasser T. (2008). Towards pervasive computing in health care: A literature review. BMC Med. Inform. Decis. Mak..

[62] Michie S., Prestwich A. (2010). Are interventions theory-based? Development of a theory coding scheme. Health Psychol..

[63] Alemdar H., Ersoy C. (2010). Wireless sensor networks for healthcare: A survey. Comput. Netw..

[64] Lyles C.R., Harris L.T., Le T., Flowers J., Tufano J., Britt D., Hoath J., Hirsch I.B., Goldberg H.I., Ralston J.D. (2011). Qualitative evaluation of a mobile phone and web-based collaborative care intervention for patients with type 2 diabetes. Diabetes Technol. Ther..

[65] Free C., Phillips G., Galli L., Watson L., Felix L., Edwards P., Patel V., Haines A. (2013). The effectiveness of mobile-health technology-based health behaviour change or disease management interventions for health care consumers: A systematic review. PLoS Med..

[66] DeSmet A., Thompson D., Baranowski T., Palmeira A., Verloigne M., De Bourdeaudhuij I. (2016). Is participatory design associated with the effectiveness of serious digital games for healthy lifestyle promotion? A meta-analysis. J. Med. Internet Res..

[67] Kato P.M. (2012). Evaluating efficacy and validating health games. Games Health Res. Dev. Clin. Appl..

[68] Bates D.W. (2002). The quality case for information technology in healthcare. BMC Med. Inform. Decis. Mak..

[69] Allen J.K., Stephens J., Dennison Himmelfarb C.R., Stewart K.J., Hauck S. (2013). Randomized controlled pilot study testing use of smartphone technology for obesity treatment. J. Obes..

[70] Vanstone M., Rewegan A., Brundisini F., Dejean D., Giacomini M. (2015). Patient Perspectives on Quality of Life With Uncontrolled Type 1 Diabetes Mellitus: A Systematic Review and Qualitative Meta-synthesis. Ont. Health Technol. Assess. Ser..

[71] Browne J.L., Ventura A., Mosely K., Speight J. (2014). ‘I’m not a druggie, I’m just a diabetic’: A qualitative study of stigma from the perspective of adults with type 1 diabetes. BMJ Open.

[72] Drummond D., Hadchouel A., Tesnière A. (2017). Serious games for health: three steps forwards. Adv. Simul..

[73] Gagnon M.P., Orruno E., Asua J., Abdeljelil A.B., Emparanza J. (2012). Using a Modified Technology Acceptance Model to Evaluate Healthcare Professionals’ Adoption of a New Telemonitoring System. Telemed. J. e-Health.

